# Preparation of VX765 sodium alginate nanogels and evaluation of their therapeutic effect via local injection on myocardial infarction in rats

**DOI:** 10.1186/s40001-024-01765-z

**Published:** 2024-03-12

**Authors:** Jianlong Liu, Qingxin Tian, Mingxiao Zhang

**Affiliations:** Department of Anesthesiology, The First Affiliated Hospital of Wenzhou Medical University, Shangcai Village, Nanbaixiang, Ouhai District, Wenzhou, 325000 Zhejiang China

**Keywords:** Myocardial infarction, Sodium alginate, Nanogel, Caspase-1 inhibitor

## Abstract

Myocardial Infarction (MI) is major cause of heart failure, highlighting the critical need for effective therapeutic strategies to improve cardiac repair. This study investigated the cardioprotective effects of VX765-coated polyethyleneimine (PEI)/sodium alginate (AG) composite nanogels (AG/PEI-VX765 NGs) in a rat model of MI. Additionally, AG-VX765 NGs and PEI-VX765 nanospheres (NPs) were synthesized and tested to compare their efficacy. MI was caused in rats by ligating the left anterior descending branch of the coronary artery, and the rats were grouped and set as Sham, MI, MI + VX765, MI + AG-VX765NGs, MI + PEI-VX765NPs, and MI + AG/PEI-VX765NGs. Results demonstrate that AG/PEI-VX765NGs were non-toxic and exhibited a sustained release of VX765. In vivo, experiments demonstrated that all treatment groups significantly enhanced cardiac function, reduced infarct size, fibrosis, and apoptosis in rats with MI, with the MI + AG/PEI-VX765NGs group exhibiting the most favorable outcomes. Our findings indicate that AG/PEI-VX765NGs represent a promising therapeutic approach for MI treatment.

**Supplementary Information:**

The online version contains supplementary material available at 10.1186/s40001-024-01765-z.

## Introduction

Cardiovascular diseases, particularly myocardial infarction (MI), are a significant health concern due to their high incidence and mortality rates [1–3]. Although therapeutic interventions such as pharmacotherapy, interventional therapy, and heart transplantation, restoring damaged myocardial tissue completely remains a formidable challenge due to the limited regenerative capacity of cardiomyocytes [2, 4]. Therefore, there is an urgent need for innovative therapeutic approaches to mitigate cardiomyocyte apoptosis, reduce the MI size, enhance left ventricular function, and prevent heart failure. It is noteworthy that MI is affecting individuals at increasingly younger ages, highlighting the critical importance of developing effective novel therapies for this condition [1–3]. Thus, the development of efficacious novel therapies for MI is of paramount significance.

Previous studies have highlighted the role of pyroptosis in various cardiovascular diseases, with the caspase-1-mediated signaling pathway identified as a key player in this process [5, 6]. The caspase-1 inhibitor VX765 has shown promise in protecting against acute myocardial ischemia reperfusion (IR) injury in rat models [7], indicating its potential as a cardiac protective therapy [8]. VX765 is an orally absorbed prodrug of the active metabolite VRT-043198, and the reported half-life of VX765 in plasma following intraperitoneal injection at a dose of 50 mg ∙kg^−1^ is 3.2 h [9]. However, the short half-life of VX765 as an oral agent, attributed to its instability in acidic conditions, poses challenges in achieving optimal bioavailability, especially in the gastric environment where degradation occurs [10]. While further clinical development reports on VX765 are awaited, addressing issues of drug degradation and loss following conventional administration remains crucial.

Nanogels (NGs) have emerged as promising carriers in biomedical areas, including drug delivery, genetic engineering, and tissue engineering [11–13]. They have a three-dimensional hydrogel structure composed of hydrophilic or amphiphilic polymer chains that are physically or chemically linked [14, 15]. In the context of myocardial therapy, intramyocardial injection of NGs offers localized treatment advantages while minimizing potential systemic effects. Several NGs, such as hydrogels facilitating the delivery of therapeutic agents, have been investigated as injectable delivery carriers for targeted treatment of MI [16, 17]. Among them, sodium alginate (AG), a natural polysaccharide, stands out due to its excellent biocompatibility, biodegradability, and favorable gel properties [18, 19]. Studies have shown that injecting calcium alginate solution cross-linked with calcium into the MI site triggers a phase transition to hydrogel at the infarct site. This temporary physical support provided by the hydrogel prevents poor cardiac remodeling and dysfunction after MI in rats [20].

Despite the significant progress in hydrogel development, challenges persist in the mechanical weaknesses of natural hydrogels and the poor biocompatibility of synthetic hydrogels [21, 22]. To address these issues, polyethyleneimine (PEI), a branched polymer with abundant end groups, was utilized as a cross-linking agent to improve the biocompatibility of nanogels [22, 23]. PEI, a cationic polymer with excellent water solubility, also holds potential advantages in drug delivery [24, 25]. The interaction between the amine groups in PEI and the carboxylate groups in the alginate moieties offers a potential mechanism for stabilizing the composite platform [25].

In this study, injectable VX765-polyethyleneimine/sodium alginate nanogels (AG/PEI-VX765 NGs) were prepared using sodium alginate nanogels (AG NGs) and PEI carrier with good biocompatibility. Additionally, AG-VX765 NGs and PEI-VX765 nanoparticles (NPs) were synthesized to assess the efficacy of different formulations of VX765 with AG or PEI. The impact of VX765 free drug and these VX765 nanomaterials on cardiac function, tissue fibrosis, cardiomyocyte apoptosis and infarct area in rats with MI was evaluated. This study laid a foundation for the potential clinical application of VX765 NGs.

## Materials and methods

### Ethics approval

The protocols involving the use of animals were approved by the Animal Policy and Welfare Committee at Wenzhou Medical University (Approval documents: wydw2023-0124). For the implementation of the experimental protocols, we adhered to the ethical principles outlined in the Guide for the Care and Use of Laboratory Animals: Eighth Edition. We have taken all necessary measures to minimize the pain and suffering of the animals.

### Synthesis and characterization of AG-VX765 NGs, PEI-VX765 NPs, and AG/PEI-VX765 NGs

**Fig. 1:**
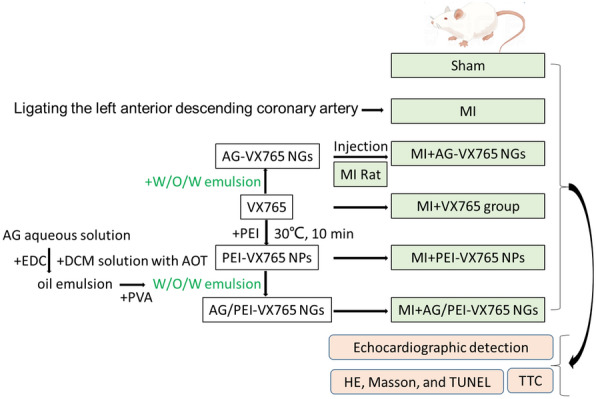
Diagram of synthesis and experimental grouping of different drug formulations

A solution of VX765-polyethyleneimine nanospheres (PEI-VX765 NP) was prepared through self-assembly [26]. PEI (branched, relative molecular weight of 600, batch number F1518083, Shanghai Aladdin Reagent Company, Shanghai, China) was dissolved in a 0.5% acetic acid solution. The VX765 drug solution (catalogue number: HY-13205; MedChemExpress, New Jersey, USA) with a concentration ranging from 0 to 1000 μM was slowly dripped into the PEI solution while stirring. The PEI-VX765 NP was obtained by stirring at a constant temperature of 30 ℃ for 10 min (Fig. 1).

AG/PEI-VX765 NGs were prepared using the double emulsification method. Initially, AG (batch number S22278, American FMC Company) was dissolved in ultra-pure water to obtain an AG aqueous solution. Subsequently, 3 mL of the AG aqueous solution was placed in a reaction bottle, followed by the addition of 8.25 mg EDC. The mixture was stirred for 3 h to activate the carboxyl group on the AG skeleton chain. Thereafter, the activated solution was dripped into DCM solution with dissolved AOT (2.5 wt%, 6 mL), and the mixed solution was stirred at 1000 rpm for 5 min to form water in oil emulsion. The oil emulsion was then dripped drop by drop into aqueous solution of PVA (45 mL 2 wt%), and the solution was stirred at 1000 rpm and mixed for 10 min to form a water-in-oil-in-water (W/O/W) emulsion. Then, the prepared aqueous solution of PEI-VX765 NPs was dropped into the W/O/W emulsion drop by drop, and the mixture was first stirred at 1000 rpm overnight for evaporation and removal of organic solvent DCM, then centrifuged at 8000 rpm, and washed three times to remove the surfactant. Finally, the product was re-dispersed in ultra-pure water or PBS buffer to obtain AG/PEI-VX765 NGs. The preparation of AG-VX765 NGs was performed using the same method as described above, except for the dripped into the above PEI solution step (Fig. 1). Fourier transform infrared (FTIR) analysis was performed to analyze the PEI and the formation of AG/PEI, which was conducted using a Bruker-IFS-48 FTIR spectrometer (Ettlingen, Germany) in the range of 500–4000 cm^−1^. Particle size and Zeta potential were determined using a nanoparticle size analyzer (Zetasizer Nano ZS90, Malvern Instruments, UK). The morphology of alginate brine gels was observed under the scanning electron microscope (SEM, S-4800; Hitachi, Tokyo, Japan).

### *Compatibility assayed through *in vitro* viability study*

Cytocompatibility of AG/PEI-VX765 NGs was assessed using H9C2 cells through CCK-8 assay. H9C2 rat cardiomyocytes were purchased from the American Type Culture Collection (Rockville, Maryland, USA) and cultured in DMEM/ high glucose (HyClone, USA) supplemented with 10% fetal bovine serum (Gibco, USA) and 1% penicillin/streptomycin (Gibco, USA) at 37 ℃ in a 5% CO_2_ environment. VX765, PEI-VX765 NPs, AG-VX765 NGs, and AG/PEI-VX765NGs with various concentrations of VX765 (0, 25, 50, 100, 200, 400, 800 and 1000 μM) were added, respectively, to the cultured cells, rinsed twice with sterilized PBS, and then soaked in the medium overnight. The cells were inoculated on the hydrogel in a 24-well plate at a density of 2 × 10^5^ cells per well. To analyze the effect of hydrogel composition on cell viability, 10% of (v/v) cell counting kit solution (CCK-8, Kumamoto, Japan) was added to the culture supernatant, and the mixture was reacted for 2 h at 37 ℃ under 5% CO_2_. The absorbance of each hole at λ = 450 nm was detected, and the cell viability was then determined.

### In vitro* drug release*

In vitro release behavior of VX765, AG-VX765 NGs, PEI-VX765 NPs and AG/PEI-VX765 NGs was investigated in phosphate-buffered saline (PBS) using a dialysis bag method. The drugs and nanomaterials were placed in the dialysis bag and suspended in PBS. Samples were collected at specific time intervals (0, 24, 48, 72, 96, and 120 h) and centrifuged at 12000 rpm for 10 min. The supernatant was then analyzed for ultraviolet absorption at a wavelength of 480 nm. Subsequently, 5 mL of PBS buffer solution was re-added to the centrifuge tube, and the mixture was incubated in a constant temperature water bath oscillator. To ensure data accuracy, each sample was measured at least three times.

### Drug loading rate of AG/PEI-VX765NGs

An appropriate quantity of AG/PEI-VX765NGs was subjected to centrifugation at 16000 rpm for 40 min at room temperature (25 ℃). Subsequently, the supernatant was filtered through a 0.45 μm pore-size filter. The quantification of VX765 was performed using a 2695 HPLC system (Waters, NY), with detection carried out at 230 nm. The drug loading rate was calculated using the following formula:

$$\text{Drug loading rate} (\%)\hspace{0.17em}=\hspace{0.17em}A\text{mount of VX765 in NGs}/\text{Amount of NGs}\hspace{0.17em}\times \hspace{0.17em}100\%$$

### Preparation of rat MI model and injection of grouping materials

Forty-eight male SPF Sprague–Dawley rats aged 6–8 weeks (weighting 250 g ± 20 g) were obtained from the Wenzhou Medical University Animal Center. Animals were fed in a standard laboratory with controlled room temperature (22 ± 1 °C), humidity (65–70%), and 12 h of light–dark cycle, while having free access to food and water. Anesthesia was performed by intraperitoneal injection of 2% pentobarbital sodium (30 mg/kg), and the anesthetized rats received ventilator-assisted respiration for small animals (inspiratory/expiratory ratio of 1/2, tidal volume of 3 mL/100 g, and respiratory rate of 50–70 beats/min). The fourth and fifth intercostal skin of the left edge of the sternum was cut laterally, and the length of the incision was about 1.5 cm. The subcutaneous tissue, pectoralis major muscle and pectoralis minor muscle were bluntly separated, and the intercostal muscle was cut. Meanwhile, the intercostal incision was opened using the eyelid device, and the pericardium was bluntly torn open to fully expose the heart. The left anterior descending coronary artery was ligated using a 6-0 silk suture positioned at the midpoint between the pulmonary artery cone and 1–2 mm below the left atrial appendage. The ligation depth was approximately 2.0 mm. Changes in color of the ventricle wall were monitored during the ligation process. A rapid transition from red to purplish white accompanied by cyanosis in the anterior wall of the left ventricle, indicated a successful surgery for the MI model. Rats that underwent MI were administered intramuscular penicillin to prevent infection. The sham group rats underwent the same surgical procedure without coronary artery ligation. In this study, rats were randomly assigned to six groups, each consisting of 8 rats, using a random number table method: Sham group, MI group, MI + VX765, MI + AG-VX765 NGs, MI + PEI-VX765 NPs group, and MI + AG/PEI-VX765 NGs group (Fig. 1). Injection of respective materials for each group was performed on 5 different sites at the edge of the MI region in rats, with a liquid injection volume of 2 μL for each site. The chest cavity was closed after the injection, followed by layer by layer suturing of muscles and skin, and removal of tracheal intubation. Three rats died during the protocol, two in the MI group and one in the MI + VX765 group. Therefore, 6 rats per group were subsequently selected for the experiment, and all measurements were performed unknowingly by the same operators.

### Echocardiographic detection

Echocardiography was performed onSD rats 28 days after the operation to assess cardiac function improvement. The rats were anesthetized with intraperitoneal injection of 2% pentobarbital sodium (30 mg/kg) and examined using Vevo2100 ultrasound (VisualSonics, Toronto, Canada). The measured parameters included left ventricular end-systolic diameter (LVIDs), left ventricular end-diastolic diameter (LVIDd), ejection fraction (EF), and short-axis shortening rate (FS). Echocardiography was utilized to identify any alterations in rat cardiac function post-operation. The selected parameters, LVIDs, LVIDd, EF, and FS, are commonly used indicators of cardiac function in both animal models and humans. By comparing these parameters pre and post-operation, the efficacy of the proposed method in enhancing cardiac function can be evaluated.

### Triphenyl tetrazolium chloride (TTC) staining

Following echocardiographic examination, euthanasia was performed by administering an overdose of pentobarbital sodium via intraperitoneal injection in accordance with the American Veterinary Medical Association (AVMA) Guidelines for the Euthanasia of Animals, 2020 Edition. The heart was harvested and cut into 2–3 mm thick slices along the sagittal section of the heart. And the slices were kept in TTC solution (37 °C, 1%, pH7.4) for 25 min, and then fixed in 10% formaldehyde solution overnight. Finally, images were taken with a digital camera, and the infarct area was analyzed using ImageJ software.

### Histological analysis

The heart tissue was fixed in 4% paraformaldehyde at 4 °C for 48 h, dehydrated in an alcohol gradient, embedded in paraffin, and sectioned into 5 μm thick slices. Hematoxylin eosin (HE) and Masson trichromatic staining were performed on the sections, with apoptosis detection carried out through TUNEL staining using a fluorescein in situ cell death detection kit (Roche). The images were taken by microscope and quantified by ImageJ software. The percentage of infarct area was calculated as follows: infarct area (%) = fibrotic area/Total area × 100%.

### Statistical analysis

Experimental data for each group were expressed by mean ± standard deviation. In all experiments, the Kruskal-Wallis test to compare the means of the different groups, followed by post-hoc pairwise comparisons using Dunn’s test. Data processing was conducted using SPSS20.0 software. *p* < 0.05 is considered statistically significant.

## Results

### Characterization and performance evaluation of NGs

**Fig. 2:**
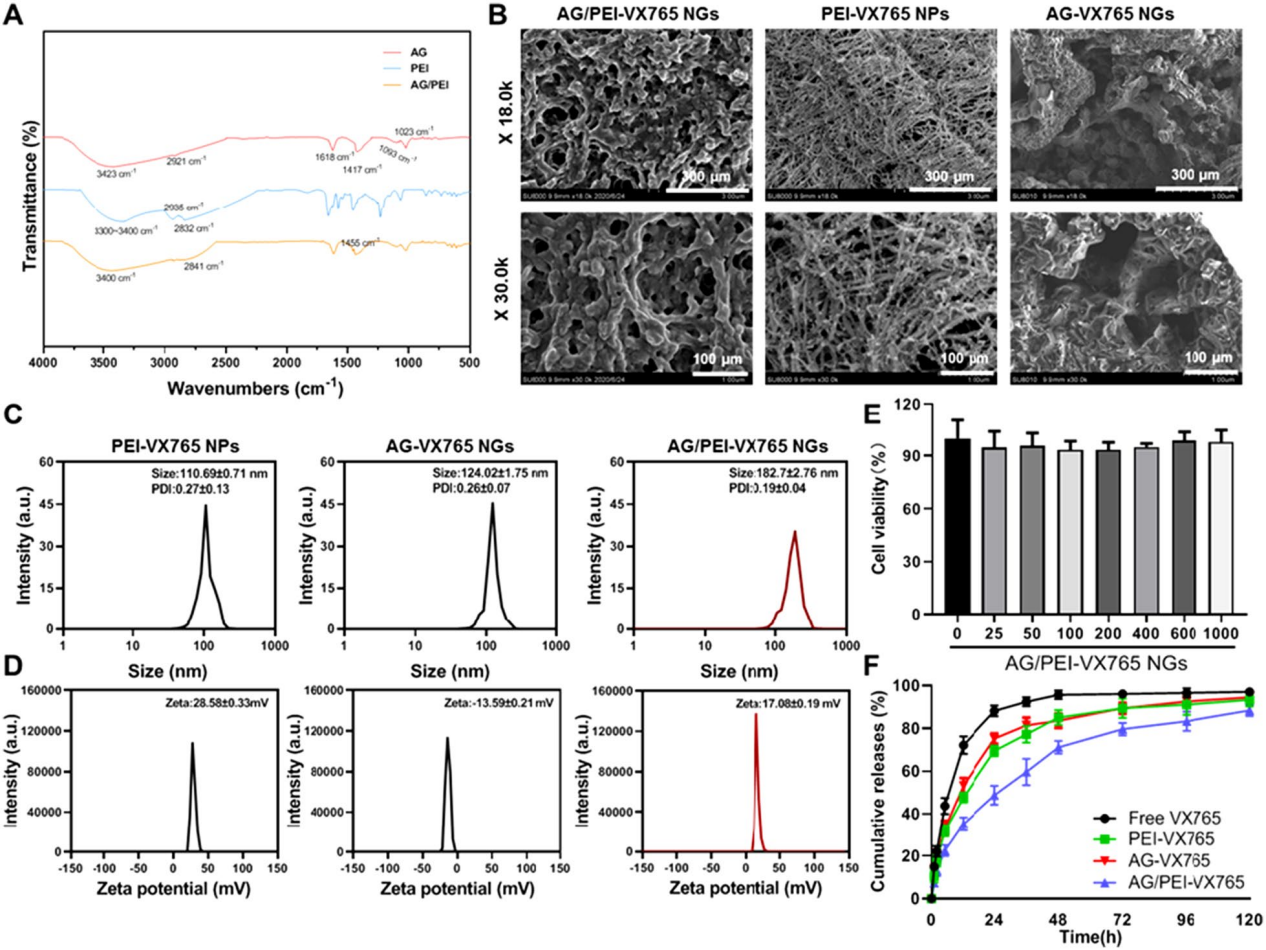
Material characterization of AG/PEI-VX765 NGs (Error bar: SD, n = 3/group). **A** FTIR spectral analysis. **B** Scanning electron microscope (SEM) test (X18.0 K, Scale Bar = 300 μm; X30.0 K, Scale Bar = 100 μm). **C** Particle sizes. **D** zeta potential. **E** CCK-8 assay-based detection of drug compatibility. **F** Drug release curves. Grouping was as follows: VX765, PEI-VX765 NPs, AG-VX765 NGs, and AG/PEI-VX765 NGs samples

Based on Fig. 2A, AG exhibits a broad absorption peak at 3423 cm^−1^, which corresponds to the stretching vibration of O−H. The peaks observed at 2921 cm^−1^ correspond to the stretching vibration of C−H, while the peaks at 1618 cm− 1 and 1417 cm^−1^ represent the asymmetric and symmetric vibrations of the carboxyl group, respectively. The peaks observed at 1093 cm^−1^ and 1023 cm^−1^ correspond to the stretching vibrations of the C−O chain in COO−and C−O−H. PEI exhibits a broad peak in the range of 3300–3400 cm^−1^, which corresponds to the stretching vibration of N–H. The peaks observed at 2935 cm^−1^ and 2832 cm^−1^ correspond to the bending vibration of methylene–CH_2_, confirming the (–CH_2_–CH_2_–NH_2_–) structure of PEI. The absorption peak observed at 3400 cm^−1^ in AG/PEI corresponds to the stretching vibrations of N–H and O–H. Additionally, a new vibration absorption peak was observed at 2841 cm^−1^, which corresponds to CH_2_, and a peak was observed at 1455 cm^−1^, which corresponds to the in-plane bending vibration of CH_2_. Overall, these findings indicate that PEI is grafted onto the AG molecular chain.

SEM images revealed the surface of AG-VX765 NGs appeared smooth, exhibiting interconnected pores, indicative of a fused thin film-like structure (Fig. 2A). Similarly, AG/PEI-VX765 NGs displayed suitable architectures with interconnected pores, forming a cohesive thin film-like structure that could support sustained VX765 release. It is noteworthy that deeper crosslinking in AG/PEI-VX765 NGs led to denser networks and enhanced mechanical strength. In contrast, PEI-VX765 NPs exhibited a branched structure with a rough surface (Fig. 2B). As presented in Fig. 2C, D, PEI-VX765 NPs exhibited an average particle size of 110.69 ± 0.71 nm, a zeta potential of 28.58 ± 0.33 mV, and a PDI value of 0.27 ± 0.13. AG-VX765 NGs had an average particle size of 124.02 ± 1.75 nm, a zeta potential of − 13.59 ± 0.21 mV, and a PDI value of 0.26 ± 0.07. AG/PEI-VX765 NGs demonstrated an average particle size of 182.7 ± 2.76 nm, a zeta potential of 17.08 ± 0.19 mV, and a PDI value of 0.19 ± 0.04. These results highlight the appropriate particle sizes and uniform size distributions of the nanogel formulations, meeting the requirements for effective drug delivery. As shown in Fig. 2E, H_9_C_2_ cells treated with AG/PEI-VX765NGs containing various concentrations of VX765 had a viability of more than 85%, indicating that the tested AG/PEI-VX765 NGs display no significant cytotoxicity but rather a good cytocompatibility. Similarly, different concentrations of VX765, PEI-VX765 NPs, and AG-VX765 NGs similarly showed good cytocompatibility (Additional file 1: Fig. S1). Moreover, we observed a relatively more persistent release of VX765 from AG/PEI-VX765 NGs compared with PEI-VX765 NPs and AG-VX765 NGs (Fig. 2F). The experimental results demonstrated that the content of VX765 in AG NGs was 6.56 ± 0.16% by HPLC analysis.

### Evaluation of cardiac function and infarct size

**Fig. 3:**
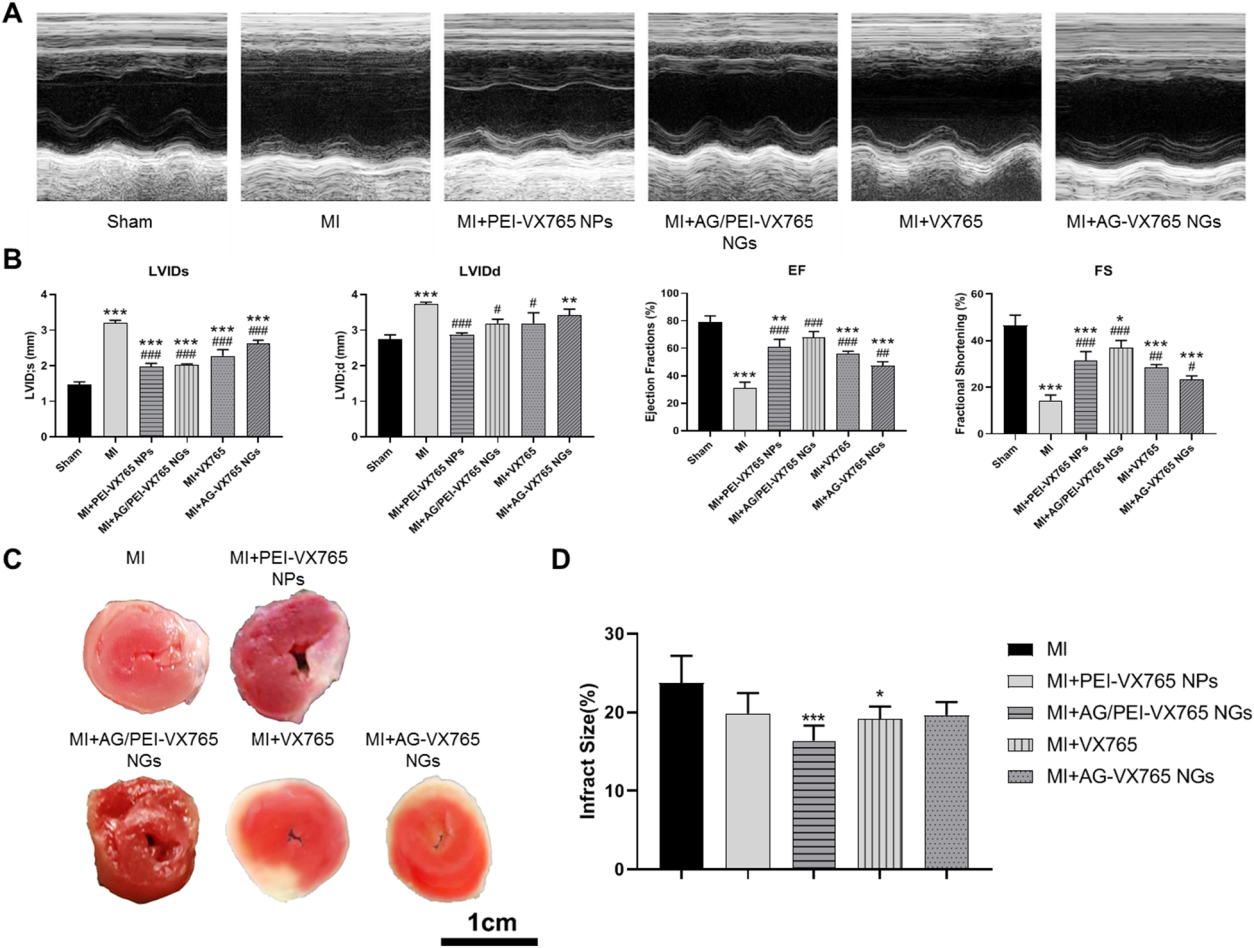
Echocardiographic and myocardial infarct size of AG/PEI-VX765 NGs (Error bar: SD, *n* = 6/group). **A** Data on M-mode echocardiogram. **B** Ultrasonic indices were shown as follows: left ventricular end-systolic diameter (LVIDs), left ventricular end-diastolic diameter (LVIDd), ejection fraction (EF), and short-axis shortening rate (FS). **C** Myocardial infarct size (Scale bar: 1 cm). **D** Myocardial infarct size was expressed as the percentage of the infarct area relative to the total at-risk area after MI (Error bar: SD). ^*^p < 0.05, and ^***^p < 0.001 compared with MI group. Grouping was as follows: MI, MI + VX765, MI + AG-VX765 NGs, MI + PEI-VX765 NPs and MI + AG/PEI-VX765NGs

Cardiac function was evaluated at the end of the study through echocardiography (Fig. 3A). As shown in Fig. 3B, the LVIDs and LVIDd of MI + VX765, MI + AG-VX765 NGs, MI + PEI-VX765 NPs and MI + AG/PEI-VX765NGs groups were significantly smaller than those in MI group (*P* < 0.05), whereas ejection fraction (EF) and short-axis shortening rate (FS) were markedly increased in the above-mentioned two groups (*P* < 0.05). Compared with the MI group, MI + VX765, MI + AG-VX765 NGs, MI + PEI-VX765 NPs, and MI + AG/PEI-VX765NGs groups exhibited a significant reduction in myocardial infarct size (*P* < 0.05, Fig. 3C, D). Among them, the MI + AG/PEI-VX765NGs group has the most significant effect.

### Histological observation

**Fig. 4:**
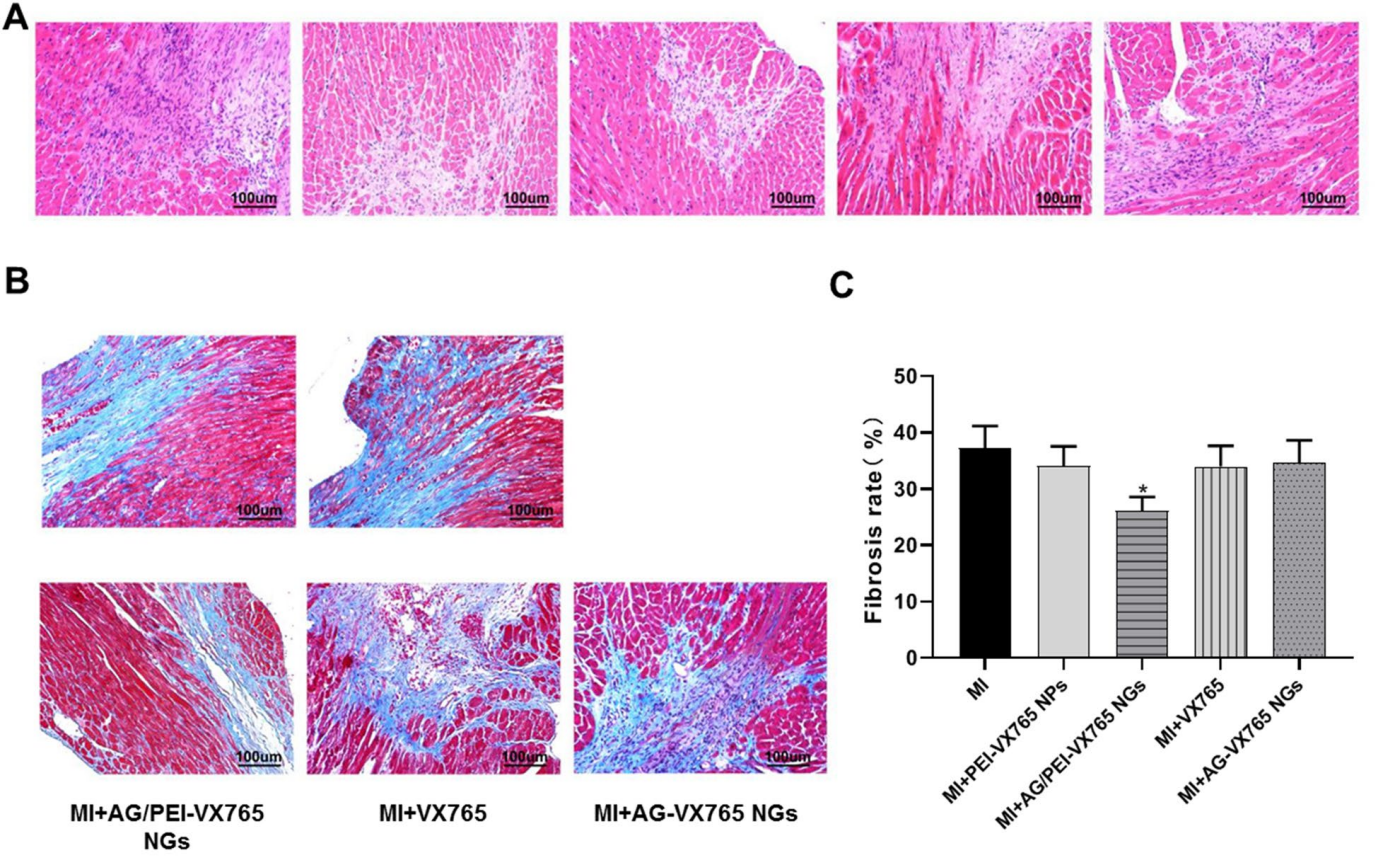
Histological observation of AG/PEI-VX765 NGs after injection (*n* = 6/group). **A** Hematoxylin and eosin (H&E) staining of myocardial tissue (Scale bar: 100 μm). **B** Myocardial section stained with Masson's trichrome (Scale bar: 100 μm). **C** Statistical analysis of Masson’s trichrome staining (Error bar: SD). ^*^p < 0.01 compared with MI group. Grouping was as follows: MI, MI + VX765, MI + AG-VX765 NGs, MI + PEI-VX765 NPs and MI + AG/PEI-VX765NGs

Fibrosis was measured by trichromatic staining of H&E and Masson 28 days after injection of AG/PEI-VX765 NGs. The histological analysis indicated a notable reduction in fibrosis in the MI + AG/PEI-VX765 NGs group compared to the MI group (*P* < 0.05, Fig. 4).

### Detection of cardiomyocyte apoptosis

**Fig. 5:**
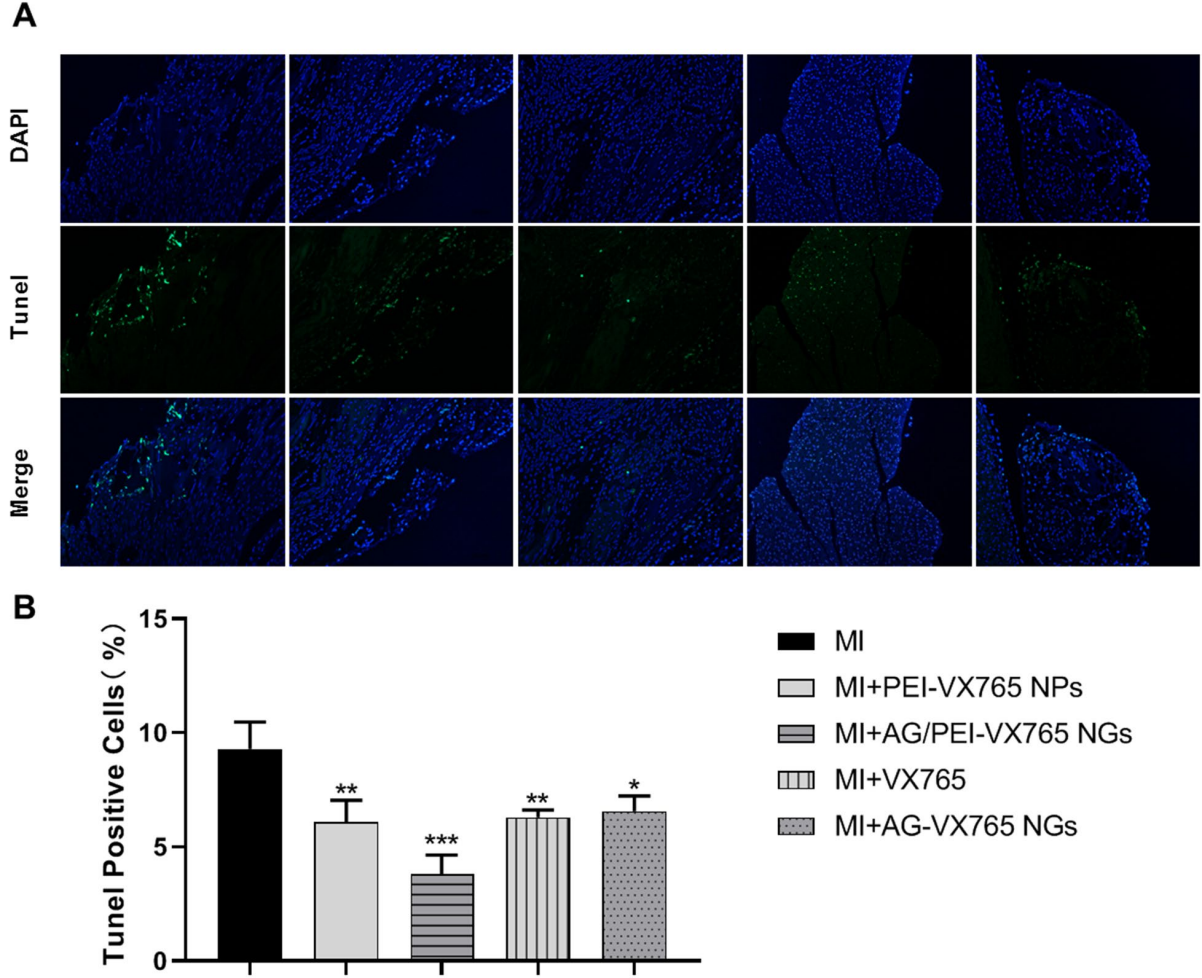
The effect of AG/PEI-VX765 NGs on myocardial apoptosis was assessed by TUNEL immunofluorescence (*n* = 6/group). **A** Microscopic observation of TUNEL-stained samples (Scale bar: 100 μm). The nucleus was stained with DAPI (blue), while apoptotic cells were visualized by TUNEL staining (green). **B** Statistical analysis of TUNEL staining (Error bar: SD). **p < 0.01 and ***p < 0.001 compared with MI group. Grouping was as follows: MI, MI + VX765, MI + AG-VX765 NGs, MI + PEI-VX765 NPs and MI + AG/PEI-VX765NGs

The TUNEL immunofluorescence assay was performed to assess apoptosis in myocardial tissue following various intervention methods. The experiments involved staining the nucleus with DAPI (blue) and visualizing apoptotic cells with TUNEL staining (green). As depicted in Fig. 5, the proportion of TUNEL positive cells significantly decreased in the MI + VX765, MI + AG-VX765 NGs, MI + PEI-VX765 NPs and MI + AG/PEI-VX765NGs groups compared to the MI group (*P* < 0.05). Notably, the AG/PEI-VX765 NGs group exhibited the lowest rate of TUNEL positive cells. These findings suggest that MI + AG/PEI-VX765 NGs could mitigate MI-induced apoptosis and safeguard cardiomyocytes.

## Discussion

Myocardial infarction (MI) is a cardiac injury disorder primarily caused by ischemia and hypoxia. Local ischemia leads to irreversible loss of cardiomyocytes, triggering inflammation, fibrosis, and cardiac dysfunction [27]. The limited regenerative and reparative capacity of mature myocardium poses a challenge for treating cardiovascular disease, as currently there are no available treatments to restore cardiac function after MI [4]. Consequently, myocardial tissue engineering has emerged as a promising approach for addressing damaged myocardium [11]. Injectable NGs provide an effective minimally invasive method to restore the development of functional myocardium, showing great significance for the treatment of cardiovascular diseases [16]. They can effectively deliver drugs to infarcted myocardial tissue, solving the major problem for the nanotherapeutic strategy, while enhancing the effect of conventional therapy [11, 16]. In this study, we prepared injectable VX765-polyethyleneimine/sodium alginate nanogels (AG/PEI-VX765 NGs). The advantages of this hydrogel in treating MI are that it can flow at low or room temperature, display favorable injectable performance. Upon injection into the body, it can rapidly transform into a gel phase, which exerts a mechanical support effect and promotes local fixation of the therapeutic agent to the myocardium. Furthermore, our findings demonstrate that AG/PEI-VX765 NGs provide a protective effect against MI, manifested by the improvement of cardiac function, reduction in cardiac fibrosis, apoptosis, and MI size.

The strategy for improving heart failure after MI by injection of biomaterials into the infarcted myocardium has proved successful [16, 17]. The composition and physicochemical properties of injected biomaterials mainly determine the repair effect in the infarcted myocardium. In this study, AG and PEI were used as the main materials. Because there are abundant carboxylic acid groups in the AG polymer chain, AG/PEI-VX765NGs is chemically cross-linked with the carboxylic acid group on AG through the surface amino group of PEIs, thereby being wrapped in AG. We observed that AG/PEI-VX765 NGs containing VX765 at concentrations ranging from 0 to 1000 μM demonstrated good cytocompatibility, laying a foundation for subsequent evaluation in vivo. This observation aligns with previous research findings. The ultraminiature iron oxide (Fe_3_O_4_) NPs, coated with PEI by Hao et al. [12], formed stable alginate (AG) NGs colloid through double emulsion, demonstrating favorable cell compatibility. Moreover, the sustained release of these NGs allows for extended interaction with cells, potentially enhancing regeneration. This sustained release effect has also been supported by previous studies [17, 28, 29].

Myocardial fibrosis after MI can induce malignant cardiovascular diseases [30]. It is of great clinical significance to determine the mechanism of myocardial fibrosis and undertake individualized targeted therapy. Fibrin-specific poly (N-isopropylacrylamide) NGs comprising core–shell colloidal hydrogels can inhibit cardiac fibrosis after IR injury [31]. Consistent with previous studies, histological analysis (HE and Masson) of cardiac tissue after MI in this study suggests that while interstitial and perivascular fibrosis is inhibited, administration with AG/PEI-VX765NGs reduces myocardial fibrosis. Moreover, echocardiography and TTC detection revealed that AG/PEI-VX765NGs treatment remarkably improves the cardiac function of rats with MI, indicating that NGs play a protective role in the heart, as shown in previous studies [17, 31].

Apoptosis is a significant mode of cell death in the early stages of MI, resulting in the loss of cardiomyocytes and other cell types, ultimately impacting ventricular remodeling and reducing cardiac function. Our study revealed that NGs reduced in vivo apoptosis. VX765, an inhibitor of caspase-1, plays a critical role in pyroptosis, an inflammatory form of caspase-1-dependent programmed cell death [5]. Pyroptosis and apoptosis are distinct forms of programmed cell death with unique mechanisms and outcomes. Although caspase-1 is mainly linked to pyroptosis, there can be interactions and overlaps between different forms of cell death. The crosstalk between pyroptosis and apoptosis, involving various caspases and signaling pathways, can be complex and dependent on the context. Moreover, VX-765 has demonstrated inhibition of the harmful death of vascular smooth muscle cells (VSMCs) in atherosclerosis, indicating that targeting caspase-1 activity could be a promising therapeutic approach for atherosclerotic cardiovascular diseases [32].

While AG/PEI-VX765 NGs exhibit promising enhancements in cardiac function, reduced apoptosis rates, and mitigated myocardial damage in myocardial infarction rats, our study has limitations. These include the absence of validation through cellular experiments and a thorough investigation of the mechanisms responsible for cardiac protection, which necessitates further in-depth exploration. Despite these limitations, AG/PEI-VX765 NGs demonstrate significant potential for advancements in myocardial infarction research and treatment. Nevertheless, unraveling the intricate mechanisms and potential clinical applications necessitates continued and extensive research efforts.

## Conclusions

In this study, we demonstrated for the first time that the injection of AG/PEI-VX765NGs promotes myocardial recovery and reduces apoptosis, cardiac fibrosis, and MI area, resulting in significant cardiac functional recovery. These results suggest that AG/PEI-VX765NGs could be a promising therapeutic potential for myocardial repair, and the in vivo application of injectable NGs may serve as an effective strategy for MI treatment.

### Supplementary Information

**Additional file 1: Figure S1. **CCK-8-based assay for drug compatibility of VX765, AG-VX765 NGs and PEI-VX765 NPs.

## Data Availability

The data that support the findings of this study are available from the corresponding author upon reasonable request.

## Abbreviations

AG: Sodium alginate
NG: Nanogel
PEI: Polyethyleneimine
MI: Myocardial infarction
SEM: Scanning electron microscope
CCK-8: Cell counting Kit-8
LVIDs: Left ventricular end-systolic diameter
LVIDd: Left ventricular end-diastolic diameter
EF: Ejection fraction
FS: Short-axis shortening rate
TTC: Triphenyl tetrazolium chloride
HE: Hematoxylin eosin
