# Supplementary figures and images for: Preparation of VX765 sodium alginate nanogels and evaluation of their therapeutic effect via local injection on myocardial infarction in rats
Source: Eur J Med Res. 2024 Mar 12;29:169. doi: 10.1186/s40001-024-01765-z (PMC10929227; doi:10.1186/s40001-024-01765-z)

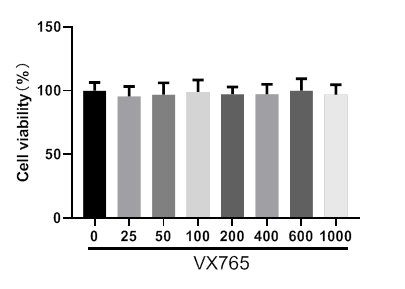


Additional file Fig. 1 CCK-8-based assay for drug compatibility of VX765, AG-VX765 NGs and PEI-VX765 NPs

Supplement: Supplementary file 1 — Additional file 1: Figure S1. CCK-8-based assay for drug compatibility of VX765, AG-VX765 NGs and PEI-VX765 NPs. [file 40001_2024_1765_MOESM1_ESM.doc]
